# Acute Effects of Single Doses of Bonito Fish Peptides and Vitamin D on Whole Blood Gene Expression Levels: A Randomized Controlled Trial

**DOI:** 10.3390/ijms20081944

**Published:** 2019-04-20

**Authors:** Frédéric Guénard, Hélène Jacques, Claudia Gagnon, André Marette, Marie-Claude Vohl

**Affiliations:** 1 School of Nutrition, Laval University, 2440 Hochelaga Blvd, Quebec, QC G1V 0A6, Canada; frederic.guenard@fsaa.ulaval.ca (F.G.); Helene.Jacques@fsaa.ulaval.ca (H.J.); 2 Institute of Nutrition and Functional Food (INAF), Laval University, 2440 Hochelaga Blvd, Quebec, QC G1V 0A6, Canada; Claudia.Gagnon@crchudequebec.ulaval.ca (C.G.); andre.marette@criucpq.ulaval.ca (A.M.); 3 Endocrinology and Nephrology Unit, CHU de Quebec Research Center, 2705 Laurier Blvd, Quebec, QC G1V 4G2, Canada; 4 Department of Medicine, Laval University, 1050 avenue de la Médecine, Quebec, QC G1V 0A6, Canada; 5 Quebec Heart and Lung Institute (IUCPQ) Research Center, 2725 chemin Sainte-Foy, Quebec, QC G1V 4G5, Canada

**Keywords:** cholecalciferol, gene expression, cardiometabolic risk, nutrigenomics

## Abstract

Fish contains high quality proteins and essential nutrients including 25-hydroxyvitamin D (25(OH)D). Fish peptide consumption can lower cardiovascular disease (CVD) risk factors, and studies have shown an association between 25(OH)D deficiency, CVD and CVD risk factors, such as diabetes. This study investigated acute effects of a single dose of cholecalciferol (VitD_3_), bonito fish peptide hydrolysate (BPH), or a combination of both on CVD risk factors and whole blood gene expression levels. A randomized, crossover, placebo controlled trial was conducted in 22 adults. They ingested, in random order and at 7-day intervals, 1000 IU of VitD_3_, 3 g of BPH, a combination of both, or a placebo. A 180 min oral glucose tolerance test was performed. Differences in whole-genome expression levels after versus before each supplementation were computed for 18 subjects. We observed that 16, 1 and 5 transcripts were differentially expressed post- vs. pre-ingestion for VitD_3_, BPH or VitD_3_ + BPH treatments, respectively. VitD_3_-containing treatments affected the expression of the solute carrier family 25 member 20 (*SLC25A20*) gene involved in fatty acid oxidation, various transcription factors and genes related to glucose metabolism. These results suggest that VitD_3_ rapidly modulates genes related to CVD risk factors in blood while BPH seems to moderately modulate gene expression levels.

## 1. Introduction

Dietary habits influence diverse cardiometabolic risk factors, including cholesterol levels, blood pressure, glucose, and insulin homeostasis [1]. Observational studies and prospective epidemiological studies reported an association between high fish consumption and reduced mortality from coronary artery disease [2,3,4]. Inverse associations between fish consumption and the risk of all-cause mortality [5,6], cardiovascular disease (CVD) [6], mortality and type 2 diabetes (T2D) [7] have been observed. Benefits from fish consumption were attributed to eicosapentaenoic acid (EPA, 20:5 (*n*-3)) and docosahexaenoic acid (DHA, 22:6 (*n*-3)) [2,3], but it was also proposed that the potential benefits of fish consumption may be related to other nutrients, such as vitamins and proteins [8,9]. Fish protein hydrolysates containing bioactive peptides were shown to possess promising properties for the prevention of CVD and risk management [9].

Regional differences were reported in the association between fish consumption and risk of mortality [6]. An inverse association between fish consumption and mortality risk was reported in Asian populations [10,11] while such an association was not confirmed in Western populations [12,13], thus, suggesting that preparation method and/or fish type may have an impact on the health outcomes of fish consumption according to geographical regions [6]. Concordant with the latter suggestions, studies on lean fish or fish protein hydrolysates demonstrated specific effects for different fish species. Dietary cod proteins were shown to improve insulin sensitivity and reduce plasma C-reactive protein (CRP) in insulin-resistant individuals [14,15]. Clinical studies demonstrated a significant reduction in serum low-density lipoprotein (LDL)-cholesterol (C) following consumption of a cod-fish protein supplement in overweight adults with no effect on triglyceride (TG) or high-density lipoprotein-cholesterol (HDL)-C levels [16]. A decrease in systolic and diastolic blood pressure (SBP and DBP) has been reported in normotensive or moderately hypertensive individuals supplemented with bonito fish [17]. Similarly, studies in animal models have demonstrated that protein sources differ in their ability to modulate CVD risk factors including insulin sensitivity [18] and lipid levels [19]. A salmon protein hydrolysate was shown to reduce glucose intolerance, dyslipidemia, and adipose tissue inflammation in obese mice fed a high-fat, high-sucrose diet [20]. Similarly, a study conducted in rats comparing various protein sources (casein and fish proteins from bonito, herring, mackerel, or salmon) in high-fat, high-sucrose diet reported a decreased expression of the pro-inflammatory cytokines TNF-α and IL-6 in fish-protein-fed groups [21], while lower weight gain, lower visceral adiposity, and improved insulin sensitivity were observed only in the salmon-protein fed group.

Low serum levels of 25-hydroxyvitamin D (25(OH)D) have been associated with mortality and deteriorated cardiovascular health reflected through unfavorable lipid profile, T2D, hypertension, and obesity [22,23,24,25]. 25(OH)D deficiency is highly prevalent in the general population, with a frequency about 30% to 50% [26] according to the generally accepted threshold level of 20 to 30 ng/mL (50–75 nmol/L) for sufficiency [27]. Discordant from the above-mentioned observations, randomized control trials (RCT) reported an increase in TG or LDL-C concentrations after cholecalciferol (VitD_3_) supplementation [28,29], which was further confirmed in a meta-analysis [30]. In view of these reports, Ponda and collaborators [29] concluded that the benefit inferred from cross-sectional associations of higher 25(OH)D levels and a healthier lipid profile was not replicated by acute VitD_3_ repletion. Similarly, RCT also showed discordant results regarding the benefits of VitD_3_ supplementation on glucose homeostasis [31,32,33].

Based on the pleiotropic effects of 25(OH)D at transcriptomic level [34] and the association of 25(OH)D deficiency with multiple diseases [22,23,24,25], RCT assessed the impact of VitD_3_ supplementation on gene expression levels. Similar to RCT on metabolic parameters, long-term supplementation studies demonstrated discordant results at gene expression levels, some studies not supporting an impact [35,36] while others reported important changes at genome-wide expression levels in healthy [37] and in metabolically deteriorated individuals [36,38].

While the risk of cardiometabolic diseases is predominantly influenced by specific foods and overall dietary patterns, assessing the impact of specific nutrients may be informative from a mechanistic point of view. Evaluating early changes in gene expression levels following nutritional treatments may reveal mediators of metabolic improvement observed under specific dietary patterns while limiting confounding factors. Therefore, the purpose of the current study was to investigate acute impacts of a single dose of VitD_3_, bonito fish peptide hydrolysate (BPH) or a combination of both, on cardiometabolic health factors and gene expression levels. A randomized, crossover, placebo controlled trial was conducted, thus, limiting inter-individual variability in the response to treatments.

## 2. Results

### 2.1. Participant Baseline Characteristics

This study included 22 Caucasian adults (11 males, 11 females) aged 23 to 68 years (mean 47.5 ± 14.2 years). Anthropometric and metabolic parameters before each treatment period did not differ between treatments (Table 1). Similar concentrations of 25(OH)D were found before each treatment period, seven to nine individuals (30–40%) showed deficiency in serum VitD_3_ levels (<20 ng/mL; <50 nmol/L). Following adjustment for age and sex, no significant associations were observed between baseline serum VitD_3_ levels and metabolic profile (Total-C, LDL-C, HDL-C, TG levels, Total-C/HDL-C ratio, blood pressure, fasting glucose and insulin, and CRP levels; *p* > 0.05 for all).

### 2.2. Changes in Glucose, Insulin, C-Peptide, and TG during Post-Oral Glucose Tolerance Test (OGTT)

There was no treatment-by-time interaction for glucose, insulin, C-Peptide, and TG levels. Differences between treatments at each time point were also tested. Moreover, no difference was identified between treatments at either of the time points (Table 2). Following adjustments for the effects of age and sex, there was no significant difference between groups in incremental areas under the curve (iAUC) for glucose (*p* = 0.97), insulin (*p* = 0.39), and C-Peptide (*p* = 0.36) levels, or in areas under the curve (AUC) for TG levels (*p* = 0.91; Supplementary Figure S1).

### 2.3. Gene Expression Analysis

**Figure 1:**
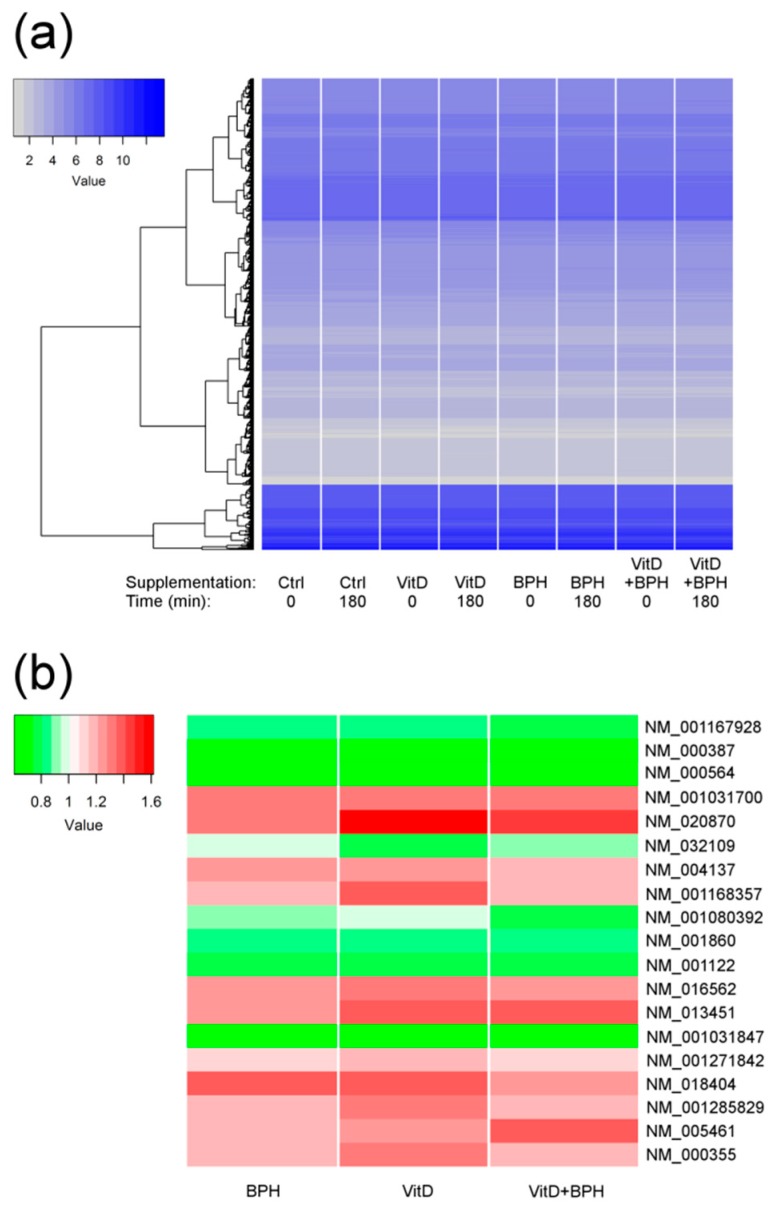
Heat maps of probe expression levels. (**a**) Heat map and hierarchical clustering of mean transcript expression levels before (Time = 0) and 3 h after (Time = 180) each treatment (24 351 expression probes). (**b**) Heat map depicting fold changes in expression levels after vs. before treatment for differentially expressed probes (false discovery rate (FDR)-adjusted *p* value ≤ 0.05).

**Figure 2:**
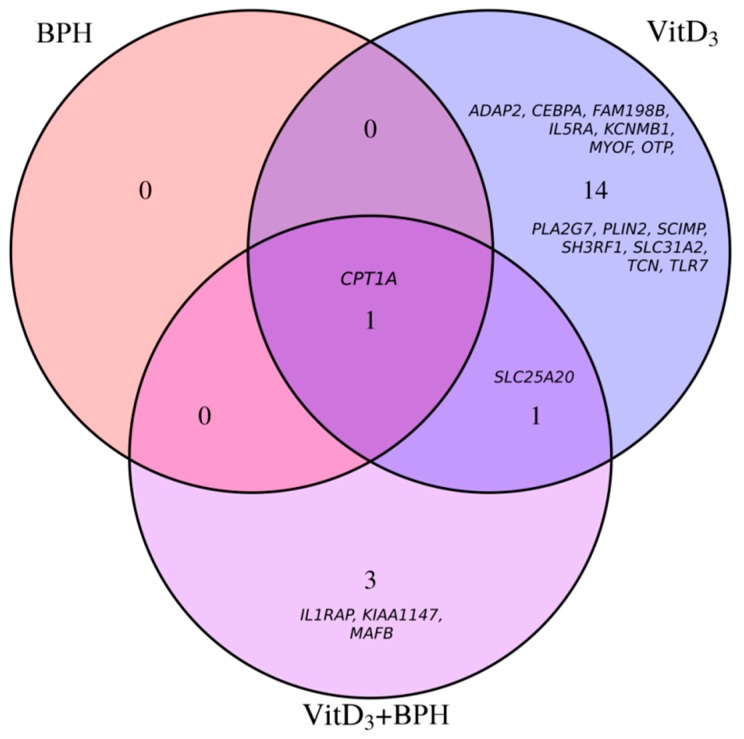
Venn diagram of treatment-regulated genes. Venn diagram showing the overlap of differentially expressed transcripts for the bonito fish peptide hydrolysate (BPH), vitamin D_3_ (VitD_3_) and the vitamin D_3_ + bonito fish peptide hydrolysate (VitD_3_ + BPH) treatments. Number of differentially expressed transcripts and gene symbols are provided.

Eighteen participants aged 24 to 68 years (mean 49.7 ± 13.7 years) were selected for further gene expression analyses. This subset composed of 10 men and 8 post-menopausal women (Table 3), did not significantly differ from the full cohort at baseline for metabolic parameters (*p* > 0.05 for all; Supplementary Table S1). Furthermore, the characteristics of the gene expression cohort did not differ between treatments at baseline. Whole-genome expression levels were measured before and 180 min post-ingestion for each treatment (placebo control, BPH, VitD_3_, VitD_3_ + BPH). Following adjustments for age, sex, BMI, and estimated blood cell count, slight differences in gene expression levels were identified for the control treatment; three transcripts (NM_002612 (pyruvate dehydrogenase kinase 4, *PDK4*), NM_001145775 (FKBP prolyl isomerase 5, *FKBP5*), NM_001015881 (TSC22 domain family member 3, *TSC22D3*)) being found differentially expressed (false discovery rate (FDR)-adjusted *p* value ≤ 0.05). These transcripts were removed from differential methylation analyses for the BPH, VitD_3_, and VitD_3_ + BPH treatments. Despite the fact that similar global expression levels were found (Figure 1), VitD_3_ treatment differentially affected 16 transcripts while BPH and VitD_3_ + BPH treatments were found to differentially change 1 and 5 transcripts, respectively (Table 4, Figure 1 and Supplementary Table S2). Among these, some transcripts were found to be differentially expressed by more than one treatment (Figure 2). A transcript for the carnitine palmitoyltransferase 1A (*CPT1A*) gene was found differentially expressed with VitD_3_, BPH, and VitD_3_ + BPH treatments. VitD_3_-containing treatments, with or without BPH, affected transcript of the solute carrier family 25 member 20 (*SLC25A20*) gene. Both genes are known to encode proteins involved in fatty acids β-oxidation. The NM_001285829 transcript, encoding the CCAAT enhancer binding protein alpha (CEBPA) transcription factor involved in cell cycle regulation and body weight homeostasis, was also found to be differentially expressed by VitD_3_ treatment.

The list of differentially expressed transcripts following VitD_3_ treatment was submitted for gene function and pathway analysis. The top two most significant overrepresented functions were Lipid metabolism and Molecular transport functions (Supplementary Table S3). Differentially expressed transcripts encoding SLP adaptor and CSK interacting membrane protein (SCIMP), CPT1, phospholipase A2 group VII (PLA2G7), perilipin 2 (PLIN2) and CEBPA proteins were common to these functions. In addition, three pathways (Mitochondrial L-carnitine Shuttle; Role of Macrophages, Fibroblasts and Endothelial Cells in Rheumatoid Arthritis; Unfolded protein response) were found overrepresented among differentially expressed transcripts following VitD_3_ treatment.

## 3. Discussion

In the current study, we used a cohort of metabolically deteriorated men and women to assess the effects of BPH, VitD_3_, and VitD_3_ + BPH treatments at the metabolic and gene expression levels. While the study was not specifically designed to include 25(OH)D-deficient individuals, the cohort included about one-third of subjects with 25(OH)D deficiency, which is similar to the prevalence reported in Canada during fall/winter where most of our recruitment took place. Our study differs from most supplementation studies as it did not aim at evaluating the long-term effects of fish peptides and/or VitD_3_ on CVD risk factors. It specifically focused on acute effects of treatments at the gene expression level. It was designed to investigate the effects of low-dose acute exposition to BPH and/or VitD_3_ in overweight or obese participants rather than to study the effects of restoring sufficiency in 25(OH)D levels in severely metabolically compromised individuals. The latter study design may increase the number of differentially expressed transcripts in comparison to numbers identified in the present study.

We conducted a randomized, crossover, placebo controlled trial in which each participant followed all treatments and the placebo treatment. While this study design minimized confounding factors, we did not observe an acute effect of 1000 IU of VitD_3_, 3g of BPH or VitD_3_ + BPH treatments on glucose homeostasis (glucose, insulin, and C-Peptide). Although the effect of BPH on glucose homeostasis and lipid profile were not studied in RCT, discordant results were observed regarding the benefits of VitD_3_ supplementation on glucose homeostasis [31,32,33]. A long-term study that tested the impact of 8-week VitD_3_ supplementation (50,000 IU weekly) on TG levels in 25(OH)D deficient individuals [29] did not report improvements in lipid profile similarly to results obtained in the current acute study. In contrast, these results are discordant with those of a recent 8-week RCT reporting increased blood lipid concentrations following high-dose VitD_3_ (2800 IU daily) supplementation in hypertensive, middle-aged patients with low 25(OH)D levels [40]. Focusing on patients with T2D, a meta-analysis of RCT identified lowering effects of VitD_3_ on Total-C and LDL-C, without significant changes in TG levels [41]. Such discrepancies between results from the above-mentioned studies may be explained by the metabolic state of the study participants, the dose administered or the duration of treatment. Moreover, 25(OH)D was shown to act on a variety of different mechanisms in addition to regulating the transcription of target genes through modulation of the activity of the vitamin D receptor [42], potentially contributing to the explanation of these discrepancies.

Specifically investigating changes in gene expression levels following acute treatment with BPH and VitD_3_, the current study was designed to explore potentially new mechanisms underlying the acute impact of such treatments. Following the removal of differentially expressed transcripts found in the placebo treatment, overlap was identified among differentially expressed transcripts from the three treatments. A decrease in *CPT1A* gene expression level was observed for all three active treatments while *SLC25A20* showed statistically significant reduction in gene expression levels under VitD_3_-containing (VitD_3_ and VitD_3_ + BPH) treatments. The *CPT1A* gene encodes a carnitine-dependent transporter involved in mitochondrial oxidation of long-chain fatty acids. It is one of the rate-limiting steps in fatty acid β-oxidation [43]. Following translocation of fatty acids through the plasma membrane, they are activated by reaction with CoA to form acyl-CoAs and thereafter, converted to acylcarnitines by mitochondrial membrane CPT1 (encoded by the *CPT1A* gene in several tissues). Acylcarnitines are then translocated into the mitochondria by solute carrier family 25 member 20 (SLC25A20), formerly termed carnitine acylcarnitine translocase (CACT). Following reconversion of acylcarnitines into acyl-CoAs, they are processed to further undergo fatty acid β-oxidation [44], thus, resulting in energy production. These two genes, herein showing reduced levels in the blood following treatment, were found among the top increased genes in the peripheral blood mononuclear cells transcriptome of diet-induced obese rats [45,46], concordant with the reported inverse association between serum 25(OH)D, weight, and fat mass [47]. Results from the current study are in accordance with results from a recent 12-week RCT (2000 IU daily) in obese subjects demonstrating an enrichment of genes related to mitochondrial function among VitD_3_ regulated genes. On the opposite, reduced expression of genes participating in fatty acid β-oxidation would potentially result in lower fatty acid β-oxidation and triacylglycerol accumulation, which is concordant with results from RCT [40,41], thus, adding to discrepancies on the potential mechanisms linking 25(OH)D and cardiovascular health [34]. In view of above-mentioned mechanisms and in combination to results from function and pathway analyses showing overrepresentation of Lipid metabolism and Molecular transport functions, our gene expression results argue for a metabolic impact of 25(OH)D possibly through modification of lipid metabolism. Changes identified here may be acutely driven, tissue-specific or dose-dependent. Based on the short-term effects of VitD_3_ herein identified, it is tempting to speculate that these effects may be mediated through membrane-based signaling rather than through vitamin D receptor signaling [42]. Specifically assessing short-term effects of a single dose of VitD_3_ may potentially limit the effects of the treatment through long-term mechanisms of action, and explain the low number of differentially expressed transcripts identified in comparisons to long term supplementation studies [36]. The low-dose, acute exposition to VitD_3_ also limits changes induced by the treatment. An important reduced number of significantly regulated genes was observed in the present study in comparison to the >700 genes reported in peripheral blood mononuclear cells, with expression levels measured 24 h after an 80,000 IU (2000 µg) VitD_3_ bolus in five participants [48]. The opposite effects of high dose VitD_3_ were recently reported in differentiated adipocytes with increased *CPT1A* gene expression, stimulated fatty acid oxidation, and reduced triacylglycerol accumulation [49].

The effect of VitD_3_ supplementation on glucose metabolism remains controversial, some studies reporting a reduction in glycemic markers and others reporting the opposite effects [7,50]. Nevertheless, 25(OH)D is thought to play a role in glycemic control through different mechanisms. It was shown to modulate insulin synthesis in pancreatic beta cells and to influence insulin sensitivity [51,52,53]. The current analysis identified overexpression of the *CEBPA* gene encoding a transcription factor (TF) belonging to the CCAAT/enhancer binding proteins (CEBP) family. This TF plays an important regulatory role in glucose metabolism, cell cycle, hematopoiesis, skeletal development, immune response, and adipocyte differentiation [54]. Based on the regulatory role of *CEBPA* in glucose metabolism, it could be hypothesized that the relationship between 25(OH)D and the glucose metabolic variables reported in RCT [31,32] may be modulated through regulation of TF and downstream targets acting on target tissues [50].

The beneficial effects of fish consumption regarding CVD mortality in regular fish consumers may be attributable to several nutrients and not only to omega-3 polyunsaturated fatty acids. Our study investigating the acute effects of fish nutrients indicate rapid onset modulation of some genes related to lipid and glucose metabolism by VitD_3_ treatment. Acute BPH treatment only slightly modulated gene expression levels in blood. Longer exposure is likely necessary to exert genomic effects. In conclusion, the present study at gene expression level shows acute modulation of lipid and glucose metabolism following VitD_3_ treatment in line with protective long-term effects reported in observational studies.

## 4. Materials and Methods

### 4.1. Study Participants

Individuals from the greater Quebec City metropolitan area were recruited through advertisements in local newspapers and by electronic messages sent to university students/employees. Subjects were recruited from September 2015 to May 2016. This study evaluated the effects of a short-term treatment on cardiometabolic risk markers, and included 11 men and 11 women (2 pre-menopausal and 9 post-menopausal) aged between 18 and 65 years old. Participants had to be overweight or obese (BMI ranging from 25 to 40 kg/m^2^) and free of any metabolic disorders requiring treatment. Individuals were excluded if they were using medications affecting glucose or vitamin D metabolism or plasma lipid levels. Volunteers who had been taking supplements in the last 3 months were excluded. Subjects were also excluded if they had fish/seafood taste aversion or allergy, were regular alcohol drinkers (>2 drinks/d), had lost >5% weight over the last 3 months, had major surgery in the 3 months prior to study onset. Among the 39 subjects screened, 24 were eligible; one chose not to participate before the beginning of the study while another withdrawn during the study for personal reasons, thus, resulting in 22 participants at the end of the study including 6 smokers.

Waist girth, resting SBP and DBP were measured using standardized procedures [55]. BMI was calculated as weight in kilograms divided by height in square meters. This study was conducted in accordance with the Declaration of Helsinki and approved by the Université Laval Ethics Committee (approval number 2015-090; approved on 1 July 2015). All participants provided written informed consent, and the trial was registered before study commencement at clinicaltrials.gov as NCT02668159.

### 4.2. Study Design

A randomized, crossover, placebo controlled trial was conducted. This study consisted of 5 visits to the Institute of Nutrition and Functional Food (INAF) clinical investigation unit. The first visit was a screening visit. Participants filled in questionnaires about socio-demographic characteristics, medical history, and lifestyle habits as well as physical activity [56]. A questionnaire documenting sun exposure in weekdays, weekends, and last vacations, as well as the use of tanning beds and sunscreen was administered. A validated food frequency questionnaire [57], documenting diet for the last month, including fish, seafood, and alcohol consumption, was administered at the first treatment visit by a registered dietitian. A medical follow-up form was completed at each visit to document changes in medical condition and medication during the study. Subjects were asked to follow the same eating habits and physical activity throughout the study, and to limit their alcohol consumption during the protocol. Two regular drinks per week were allowed, but alcohol abstinence was required 48 h before study visits. In addition, subjects were not allowed to take omega-3, VitD_3_, multivitamin or protein supplements during the study. They were also asked to avoid any natural health or omega-3 enriched products, and to limit fish and seafood consumption to 2 servings per week during the protocol. Subjects were asked to report any deviation during the protocol.

### 4.3. VitD_3_ and BPH Treatment

Following the screening visit, participants were randomized to a treatment sequence composed of placebo, BPH, VitD_3_, and VitD_3_ + BPH treatment in random order using single randomization generator implemented in INAF online platform for managing nutritional clinical studies. Participants ingested, in random order and at 7-day intervals, 1000 IU (25 µg) of VitD_3_ (cholecalciferol; Santé Naturelle A.G. Ltée, Brossard, QC, Canada), 3 g of BPH (PeptACE^®^, Natural Factors Nutritional Products Limited, Coquitlam, BC, Canada), a combination of both, or a placebo. Study capsules were coded by a third party. A registered dietitian randomized the treatments and observed the consumption of the capsules by the participants. Participants were blinded to the treatment. All treatments consisted of 7 capsules, thus, resulting in similar-looking treatments despite slight differences for the BPH capsules. Participants, thus, consumed either a placebo (7 capsules of lactose), fish peptides (6 PeptACE^®^ Fish Peptides capsules + 1 lactose capsule), VitD_3_ (1 VitD_3_ capsule + 6 lactose capsules), a combination of fish peptides and VitD_3_ (6 PeptACE^®^ capsules + 1 VitD_3_ capsule) right before an OGTT.

### 4.4. Blood Sampling and Biochemical Measures

A research nurse collected blood samples from an antecubital vein into vacutainer tubes containing ethylenediaminetetraacetic acid following a 12-h overnight fast and 48-h alcohol abstinence. Blood samples were collected during screening (visit 1) to identify and exclude individuals with any metabolic disorder as well as prior to each treatment (visits 2–5). Plasma samples were immediately processed after blood collection, aliquoted, and stored at −80°C. Plasma lipids (Total-C, LDL-C, HDL-C, and TG), as well as fasting plasma glucose and insulin concentrations, were measured using standardized procedures. Plasma CRP was measured by nephelometry (Prospec equipment, Behring, Siemens Healthcare Diagnosis, Deerfield, IL, USA) using a sensitive assay [58]. Fasting plasma insulin and glucose were measured by radioimmunoassay with polyethylene glycol separation, and enzymatically measured, respectively. Serum 25(OH)D (25-hydroxyvitamin D) levels were measured by electrochemiluminescence (Cobas System, Roche, Laval, QC, Canada) before each treatment. A 180-min OGTT (75 g of glucose) was performed following each treatment. Blood samples were collected into vacutainer tubes containing EDTA at 0, 15, 30, 60, 120, and 180 min, and glucose, insulin, C-peptide were measured. TG levels during an OGTT, being reported to be higher in insulin-resistant obese individuals and associated with metabolic risk, were also monitored throughout OGTT [59].

### 4.5. Gene Expression Analysis

A subset of 18 participants representative of the full cohort was selected for gene expression analyses. More precisely, this subset was selected to exclude pre-menopausal women to avoid the confounding effect of fluctuations in estrogen levels on serum 25(OH)D levels [60]. Blood samples were collected from an antecubital vein into PAXgene Blood RNA Tubes (Qiagen, Mississauga, ON, Canada) before and 180 min after each treatment. Total RNA was isolated and purified from blood samples using PAXgene Blood RNA Kit (Qiagen, Valencia, CA, USA)) according to the manufacturer’s instructions. Quality and integrity of the purified RNA were assessed using both the NanoDrop (Thermo Scientific, Wilmington, DE, USA) and the 2100 Bioanalyzer (Agilent Technologies, Cedar Creek, TX, USA). Among the 144 RNA samples extracted (18 individuals, 4 treatments, 2 times), three samples did not meet quality criterion and were excluded from expression analyses. Expression levels were measured using the Affymetrix Clariom S HT (Thermo Fisher Scientific Inc., Waltham, MA, USA) and processed at the McGill University and Genome Quebec Innovation Centre (Montreal, QC, Canada). Arrays were scanned on an Agilent GeneArray Scanner (Thermo Fisher Scientific Inc.), and raw data were extracted from scanned images (CEL files) using Expression Console (v 1.4; Thermo Fisher Scientific Inc.). Expression Console was used to analyze image data and perform quality control. The Robust Multiarray Analysis method was used to perform background correction and quantile normalization. The Transcriptome Analysis Console Software (v2.0; Thermo Fisher Scientific Inc.) was used to extract normalized gene expression data from CHP files. Data reported in this paper have been deposited in the Gene Expression Omnibus (GEO) database, www.ncbi.nlm.nih.gov/geo (accession number GSE129604). Whole-genome expression levels were, thus, measured before and 180 min post-ingestion for each treatment (Placebo control (17 before, 18 at 180 min), BPH (18 before and at 180 min), VitD_3_ (17 before, 18 at 180 min), VitD_3_ + BPH (18 before, 17 at 180 min)). Following background correction and quantile normalization, blood cell proportions were predicted for each sample based on gene expression levels using the CellMix package (v1.6) [61]. Estimated cell counts were obtained and grouped in three classes: lymphocytes, neutrophils, and monocytes. Transcript levels were, thereafter, corrected for age, sex, BMI, serum 25(OH)D levels and estimated blood cell count. A moderated paired *t*-test on residuals was computed to test differences in expression levels after versus before treatment using the linear modeling approach and empirical Bayes statistics implemented in the limma software (v3.37.1) [62]. Changes observed in placebo (after vs. before treatment), possibly induced by venipuncture or OGTT, were systematically excluded from results obtained with other treatments.

Analysis of functions and pathways enriched from the list of differentially expressed genes following VitD_3_ treatment was conducted using the knowledge base of the Ingenuity Pathway Analysis system^®^ (Ingenuity Systems, Redwood City, CA, USA).

### 4.6. Statistics

Clinical data before each treatment were expressed as mean ± standard deviation. Group differences at baseline and at each time of the OGTT were tested using analysis of variance (general linear model). Comparisons among groups were performed using Duncan’s multiple range test when differences were identified. Treatment-by-time interaction effects on dependent variables were assessed by using MIXED procedures for repeated measurements. Difference in 25(OH)D deficiency (25(OH)D <20 ng/mL; <50 nmol/L) [26] between each treatment phases were tested using Chi-squared test. AUC (TG) and the iAUC (glucose, insulin, and C-peptide) were calculated for the 180 min OGTT interval using the trapezoidal method. Statistical analyses on clinical data were performed using SAS statistical package version 9.4 (SAS Institute Inc., Cary, NC, USA).

## Figures and Tables

**Table 1:** Pre-treatment participants’ characteristics.

Characteristics	Control	BPH	VitD_3_	VitD_3_ + BPH	*p* Value ^†^
Number of subjects (males/females)	22 (11/11)	22 (11/11)	22 (11/11)	22 (11/11)	---
BMI (kg/m^2^)	31.5 ± 4.9	31.4 ± 4.9	31.4 ± 4.8	31.4 ± 4.8	1.00
Waist girth (cm)	103.8 ± 14.5	103.5 ± 14.5	103.7 ± 14.4	103.6 ± 14.3	1.00
**Lipid profile**					
Total-C (mmol/L)	4.93 ± 0.98	5.09 ± 0.85	5.18 ± 0.86	5.17 ± 0.89	0.78
LDL-C (mmol/L)	2.95 ± 0.74	3.10 ± 0.76	3.04 ± 0.61	3.13 ± 0.72	0.84
HDL-C (mmol/L)	1.26 ± 0.28	1.30 ± 0.33	1.35 ± 0.38	1.27 ± 0.34	0.83
TG (mmol/L)	1.56 ± 0.67	1.50 ± 0.59	1.74 ± 1.07	1.81 ± 1.21	0.89
Total-C/HDL-C	4.07 ± 1.16	4.13 ± 1.19	4.14 ± 1.39	4.32 ± 1.32	0.92
**Blood pressure (mm Hg)**					
SBP	114.1 ± 8.6	112.2 ± 8.0	112.9 ± 7.3	112.5 ± 8.3	0.88
DBP	71.3 ± 5.0	69.4 ± 7.0	71.0 ± 6.7	70.4 ± 7.6	0.77
**Glucose homeostasis**					
Fasting glucose (mmol/L)	5.13 ± 0.46	5.16 ± 0.42	5.25 ± 0.43	5.19 ± 0.46	0.84
Fasting insulin (pmol/L)	114.0 ± 54.3	118.8 ± 61.5	115.8 ± 42.9	116.0 ± 53.5	0.99
HOMA-IR	3.74 ± 1.83	3.91 ± 2.05	3.87 ± 1.44	3.85 ± 1.80	0.99
**Diabetes status (Nondiabetic/prediabetic/de novo diabetes)**					
Fasting glucose	21/1/0	21/1/0	21/1/0	21/1/0	1.00
2 h, 75 g OGTT	16/6/0	---	---	---	---
CRP (mg/L)	2.86 ± 3.58	3.27 ± 3.52	2.58 ± 2.65	2.76 ± 4.03	0.95 *
25-hydroxyvitamin D (nmol/L)	56.6 ± 19.8	57.3 ± 21.9	56.8 ± 20.3	57.1 ± 19.1	1.00
Deficiency/sufficiency	8/14	9/13	9/13	7/15	0.91 ^‡^

Values presented (means ± SD) are untransformed and unadjusted values. Diabetes status defined according to fasting and 2 h post-oral glucose tolerance test (OGTT) glucose levels [39]. 25-hydroxyvitamin D (25(OH)D) deficiency defined as serum levels <50 nmol/L (<20 ng/mL). * *p* value obtained from log_10_-transformed values. ^†^ *p* value from analysis of variance (ANOVA) for between-group differences. ^‡^ *p* value obtained from Chi^2^ test. Abbreviations: BPH, Bonito fish peptide hydrolysate treatment; VitD_3_, vitamin D_3_ treatment; BMI, body mass index; Total-C, total cholesterol; LDL-C, low-density lipoprotein cholesterol; HDL-C, high-density lipoprotein cholesterol; TG, triglycerides; SBP, systolic blood pressure; DBP, diastolic blood pressure; OGTT, oral glucose tolerance test; CRP, C-reactive protein.

**Table 2:** Effects of treatments on glucose, insulin, C-peptide and triglyceride during oral glucose tolerance test.

Treatment Metabolic Parameter	0	15	30	60	120	180	*p* Value ^†^
**Control**							
Glucose (mmol/L)	5.13 ± 0.46	6.86 ± 0.97	7.99 ± 1.60	7.72 ± 2.58	6.14 ± 1.64	4.40 ± 1.35	0.98
Insulin (pmol/L)	114.0 ± 54.3	524.0 ± 286.5	858.6 ± 446.3	977.5 ± 502.9	733.6 ± 402.5	286.5 ± 258.7	0.21
C-peptide (pmol/L)	894.7 ± 279.9	2028.3 ± 659.5	3016.9 ± 944.6	3811.3 ± 1047.0	3624.4 ± 1169.0	2171.6 ± 944.1	0.41
TG (mmol/L)	1.43 ± 0.67	1.40 ± 0.60	1.47 ± 0.63	1.52 ± 0.64	1.41 ± 0.67	1.37 ± 0.65	0.54
**BPH**							
Glucose (mmol/L)	5.16 ± 0.42	7.06 ± 1.11	8.08 ± 1.79	7.61 ± 2.31	6.51 ± 1.87	4.39 ± 1.80	
Insulin (pmol/L)	118.8 ± 61.5	592.2 ± 398.1	1025.9 ± 565.4	1151.6 ± 872.5	947.8 ± 506.2	313.5 ± 276.5	
C-peptide (pmol/L)	960.0 ± 287.7	2284.0 ± 889.1	3382.1 ± 1091.7	4163.0 ± 1467.1	4189.3 ± 1163.2	2487.0 ± 1034.6	
TG (mmol/L)	1.36 ± 0.56	1.38 ± 0.56	1.40 ± 0.54	1.42 ± 0.57	1.36 ± 0.55	1.36 ± 0.56	
**VitD_3_**							
Glucose (mmol/L)	5.25 ± 0.43	7.00 ± 0.94	8.08 ± 1.42	8.03 ± 2.67	6.49 ± 1.97	4.41 ± 1.61	
Insulin (pmol/L)	115.8 ± 42.9	562.0 ± 309.1	898.6 ± 469.9	1052.1 ± 495.2	914.6 ± 527.0	300.9 ± 252.6	
C-peptide (pmol/L)	934.4 ± 225.8	2132.8 ± 682.2	3169.7 ± 957.7	4083.3 ± 899.8	4016.3 ± 1448.1	2282.7 ± 1075.2	
TG (mmol/L)	1.59 ± 0.99	1.58 ± 0.98	1.58 ± 0.96	1.59 ± 0.94	1.53 ± 0.97	1.54 ± 0.99	
**VitD_3_ + BPH**							
Glucose (mmol/L)	5.19 ± 0.46	7.21 ± 1.13	7.90 ± 1.65	8.12 ± 2.49	6.42 ± 1.78	4.39 ± 1.48	
Insulin (pmol/L)	116.0 ± 53.5	624.5 ± 351.7	889.6 ± 595.9	1114.2 ± 772.1	1035.7 ± 508.3	393.7 ± 372.3	
C-peptide (pmol/L)	957.9 ± 292.1	2295.3 ± 783.2	3179.8 ± 1071.9	4177.2 ± 1195.0	4229.2 ± 1106.9	2478.3 ± 1264.5	
TG (mmol/L)	1.61 ± 0.99	1.62 ± 0.97	1.59 ± 0.85	1.60 ± 0.83	1.45 ± 0.81	1.46 ± 0.69	
Glucose *p* value ^††^	0.81	0.71	0.98	0.88	0.89	1.00	
Insulin *p* value ^††^	0.99	0.78	0.70	0.84	0.23	0.63	
C-peptide *p* value ^††^	0.83	0.58	0.68	0.71	0.35	0.70	
TG *p* value ^††^	0.69	0.72	0.80	0.84	0.90	0.82	

Values presented (means ± SD) are untransformed and unadjusted values. Vitamin D deficiency defined as serum levels <50 nmol/L (<20 ng/mL). ^†^ *p* value for time x treatment interaction with adjustment for age, sex, and serum 25-hydroxyvitamin D concentrations. **^††^** *p* values for between-treatment differences at each timepoint obtained from the analysis of variance (ANOVA) and adjusted for the effects of age and sex. Abbreviations: BPH, Bonito fish peptide hydrolysate treatment: VitD_3_, vitamin D_3_ treatment; TG, triglycerides.

**Table 3:** Pre-treatment characteristics for the gene expression cohort.

Characteristics	Control	BPH	VitD_3_	VitD_3_ + BPH	*p* Value ^†^
Number of subjects (males/females)	18 (10/8)	18 (10/8)	18 (10/8)	18 (10/8)	---
BMI (kg/m^2^)	31.4 ± 4.9	31.3 ± 4.9	31.3 ± 4.9	31.3 ± 4.8	1.00
Waist girth (cm)	105.7 ± 14.9	105.3 ± 15.0	105.6 ± 14.9	105.5 ± 14.8	1.00
**Lipid profile**					
Total-C (mmol/L)	4.90 ± 0.95	5.14 ± 0.89	5.25 ± 0.89	5.18 ± 0.94	0.68
LDL-C (mmol/L)	2.95 ± 0.68	3.17 ± 0.77	3.15 ± 0.58	3.18 ± 0.72	0.70
HDL-C (mmol/L)	1.23 ± 0.25	1.26 ± 0.27	1.33 ± 0.35	1.27 ± 0.29	0.76
TG (mmol/L)	1.56 ± 0.70	1.54 ± 0.62	1.66 ± 1.06	1.59 ± 0.82	0.98
Total-C/HDL-C	4.09 ± 1.05	4.24 ± 1.14	4.18 ± 1.38	4.24 ± 1.12	0.98
**Blood pressure (mm Hg)**					
SBP	115.5 ± 8.7	113.2 ± 8.2	114.6 ± 6.9	113.6 ± 8.6	0.83
DBP	70.9 ± 4.9	68.5 ± 7.1	70.7 ± 7.1	69.1 ± 7.2	0.64
**Glucose homeostasis**					
Fasting glucose (mmol/L)	5.18 ± 0.45	5.22 ± 0.43	5.29 ± 0.42	5.24 ± 0.48	0.91
Fasting insulin (pmol/L)	110.9 ± 58.6	111.0 ± 64.1	113.3 ± 45.0	108.8 ± 55.2	1.00
HOMA-IR	3.71 ± 2.02	3.73 ± 2.20	3.82 ± 1.49	3.67 ± 1.91	1.00
**Diabetes status (Nondiabetic/prediabetic/de novo diabetes)**					
Fasting glucose	18/1/0	18/1/0	18/1/0	18/1/0	1.00
2 h, 75 g OGTT	13/6/0	---	---	---	---
CRP (mg/L)	3.16 ± 3.99	3.14 ± 3.42	2.71 ± 2.92	2.98 ± 4.40	0.97 *
25-hydroxyvitamin D (nmol/L)	61.0 ± 18.5	62.2 ± 20.3	61.4 ± 18.6	61.8 ± 17.0	0.98
Deficiency/sufficiency	5/13	6/12	6/12	4/14	0.74 ^‡^

Values presented (means ± SD) are untransformed and unadjusted values. Diabetes status defined according to fasting and 2 h post-OGTT glucose levels [39]. 25(OH)D deficiency defined as serum levels <50 nmol/L (<20 ng/mL). ^†^ *p* value from analysis of variance (ANOVA) for between-group differences. * *p* value obtained from log_10_-transformed values. ^‡^ *p* value obtained from Chi^2^ test. Abbreviations: BPH, Bonito fish peptide hydrolysate treatment: VitD_3_, vitamin D_3_ treatment; BMI, body mass index; Total-C, total cholesterol; LDL-C; low-density lipoprotein cholesterol; HDL-C, high-density lipoprotein cholesterol; TG, triglycerides; SBP, systolic blood pressure; DBP, diastolic blood pressure; CRP, C-reactive protein.

**Table 4:** Differentially expressed transcripts for the BPH, VitD_3_ and VitD_3_ + BPH treatments.

Transcript	Gene Symbol	Probe ID	Treatment	Fold Change	FDR *p* Value
NM_001031847	*CPT1A*	TC1100011395.hg.1	VitD_3_	0.67	2.6 × 10^−6^
NM_001031847	*CPT1A*	TC1100011395.hg.1	BPH	0.67	0.0005
NM_001031847	*CPT1A*	TC1100011395.hg.1	VitD_3_ + BPH	0.60	0.002
NM_000387	*SLC25A20*	TC0300011038.hg.1	VitD_3_ + BPH	0.61	0.0002
NM_000387	*SLC25A20*	TC0300011038.hg.1	VitD_3_	0.63	0.009
NM_001167928	*IL1RAP*	TC0300009855.hg.1	VitD_3_ + BPH	0.78	0.04
NM_001080392	*KIAA1147*	TC0700012836.hg.1	VitD_3_ + BPH	0.81	0.04
NM_005461	*MAFB*	TC2000009116.hg.1	VitD_3_ + BPH	1.40	0.04
NM_020870	*SH3RF1*	TC0400012378.hg.1	VitD_3_	1.62	0.008
NM_001031700	*FAM198B*	TC0400012245.hg.1	VitD_3_	1.34	0.008
NM_018404	*ADAP2*	TC1700012226.hg.1	VitD_3_	1.37	0.008
NM_000564	*IL5RA*	TC0300013923.hg.1	VitD_3_	0.74	0.008
NM_001285829	*CEBPA*	TC1900010386.hg.1	VitD_3_	1.34	0.009
NM_001860	*SLC31A2*	TC0900008482.hg.1	VitD_3_	0.84	0.02
NM_001271842	*SCIMP*	TC1700009556.hg.1	VitD_3_	1.20	0.02
NM_016562	*TLR7*	TC0X00006625.hg.1	VitD_3_	1.30	0.03
NM_013451	*MYOF*	TC1000011445.hg.1	VitD_3_	1.40	0.03
NM_004137	*KCNMB1*	TC0500012791.hg.1	VitD_3_	1.24	0.03
NM_001168357	*PLA2G7*	TC0600011953.hg.1	VitD_3_	1.39	0.04
NM_032109	*OTP*	TC0500011211.hg.1	VitD_3_	0.79	0.04
NM_001122	*PLIN2*	TC0900012212.hg.1	VitD_3_	0.76	0.04
NM_000355	*TCN*	TC2200009257.hg.1	VitD_3_	1.30	0.04

List of differentially expressed transcripts (time = 180 vs. time = 0) according to treatments. Transcripts differentially expressed in more than one treatment are shown at the top of the table and separated by dotted lines. Abbreviations: FDR *p* value, False discovery rate-adjusted *p* value; BPH, Bonito fish peptide hydrolysate treatment; VitD_3_, vitamin D_3_ treatment.

## Supplementary Materials

Supplementary materials can be found at https://www.mdpi.com/1422-0067/20/8/1944/s1. Figure S1. Histograms of areas under the curve (AUC; white) and incremental AUC (iAUC; grey) for the 180 min OGTT interval. AUC and iAUC calculated from 21 participants due to missing data for a participant at 15 min under control treatment. iAUC for glucose (upper left), insulin (upper right), C-peptide (lower left) and AUC for triglyceride levels (lower right) are shown. Abbreviations: Ctrl, control treatment; BPH, Bonito fish peptide hydrolysate treatment: VitD, vitamin D_3_ treatment. Table S1. Differences in metabolic parameters between the expression subset and the full cohort. Table S2. Gene expression analysis for transcripts showing differential gene expression in at least one treatment. Table S3. Biological functions identified from the list of differentially expressed transcripts following VitD_3_ treatment.

## Abbreviations

25(OH)D	25-hydroxyvitamin D
ANOVA	Analysis of variance
AUC	Areas under the curve
BMI	Body mass index
BPH	Bonito fish peptide hydrolysate
C	Cholesterol
CACT	Carnitine acylcarnitine translocase
CEBPA	CCAAT enhancer binding protein alpha
*CPT1A*	Carnitine palmitoyltransferase 1A
CRP	C-reactive protein
CVD	Cardiovascular disease
DBP	Diastolic blood pressure
DHA	Docosahexaenoic acid
EPA	Eicosapentaenoic acid
FDR	False discovery rate
HDL-C	High-density lipoprotein cholesterol
iAUC	Incremental areas under the curve
INAF	Institute of Nutrition and Functional Food
LDL-C	Low-density lipoprotein cholesterol
OGTT	Oral glucose tolerance test
PLA2G7	Phospholipase A2 group VII
PLIN2	Perilipin 2
RCT	Randomized controlled trial
SBP	Systolic blood pressure
SCIMP	SLP adaptor and CSK interacting membrane protein
*SLC25A20*	Solute carrier family 25 member 20
T2D	Type 2 diabetes
TG	Triglyceride
TF	Transcription factor
Total-C	Total cholesterol
VitD_3_	cholecalciferol
