# Supplementary material for: Acute Effects of Single Doses of Bonito Fish Peptides and Vitamin D on Whole Blood Gene Expression Levels: A Randomized Controlled Trial
Source: Int J Mol Sci. 2019 Apr 20;20(8):1944. doi: 10.3390/ijms20081944 (PMC6514567; doi:10.3390/ijms20081944)
Supplement: Supplementary file 1 [file ijms-20-01944-s001.zip › Guenard-SuppTableS1-DiffSubsetVsFullCohort_Bonito_20190411.docx]

**Table S1.** Differences in metabolic parameters between the expression subset and the full cohort.

| **Variable** | **Screening** | | | |
| --- | --- | --- | --- | --- |
| Age | 0.63 | | | |
| Menopause | 0.49^†^ | | | |
| Sex | 0.76^†^ | | | |
| Smoking status | 0.74^†^ | | | |
|  | **Control** | **BPH** | **VitD_3_** | **VitD_3_+BPH** |
| BMI (kg/m^2^) | 0.94 | 0.93 | 0.95 | 0.93 |
| Waist girth (cm) | 0.68 | 0.69 | 0.69 | 0.68 |
| Lipid profile |  |  |  |  |
| Total-C (mmol/l) | 0.91 | 0.87 | 0.82 | 0.96 |
| LDL-C (mmol/l) | 0.98 | 0.78 | 0.54 | 0.83 |
| HDL-C (mmol/l) | 0.73 | 0.67 | 0.91 | 0.99 |
| TG (mmol/l) | 0.98 | 0.83 | 0.81 | 0.52 |
| Total-C / HDL-C | 0.95 | 0.78 | 0.92 | 0.83 |
| Blood pressure (mm Hg) |  |  |  |  |
| SBP | 0.60 | 0.70 | 0.47 | 0.68 |
| DBP | 0.80 | 0.70 | 0.89 | 0.61 |
| Glucose homeostasis |  |  |  |  |
| Fasting glucose (mmol/l) | 0.68 | 0.64 | 0.72 | 0.72 |
| Fasting insulin (pmol/l) | 0.87 | 0.70 | 0.86 | 0.68 |
| HOMA-IR | 0.96 | 0.78 | 0.91 | 0.76 |
| CRP (mg/L)* | 0.90 | 0.86 | 0.96 | 0.94 |
| Diabetes status (Nondiabetic / prediabetic / *de novo* diabetes) |  |  |  |  |
| Fasting glucose | 1.00 | 1.00 | 1.00 | 1.00 |
| 25-hydroxyvitamin D (nmol/l) | 0.47 | 0.47 | 0.47 | 0.42 |
| Deficiency^†^ | 0.74 | 0.75 | 0.75 | 0.72 |

P values for differences in metabolic parameters obtained from comparisons of the subset of 18 participants to the full cohort (22 participants) are presented. Diabetes status defined according to fasting and 2h post-OGTT glucose levels [34]. 25(OH)D deficiency defined as serum levels <50 nmol/L (<20ng/ml). ^†^ P values for categorical variables were obtained using Fisher’s test. * P values obtained from log_10_-transformed values. Abbreviations: BPH, Bonito fish peptide treatment: VitD_3_, vitamin D_3_ treatment; BMI, body mass index; Total-C, total cholesterol; LDL-C; low-density lipoprotein cholesterol; HDL-C, high-density lipoprotein cholesterol; TG, triglycerides; SBP, systolic blood pressure; DBP, diastolic blood pressure; CRP, C-reactive protein. * *P* value obtained from log_10_-transformed values.
