# Supplementary material for: Acute Effects of Single Doses of Bonito Fish Peptides and Vitamin D on Whole Blood Gene Expression Levels: A Randomized Controlled Trial
Source: Int J Mol Sci. 2019 Apr 20;20(8):1944. doi: 10.3390/ijms20081944 (PMC6514567; doi:10.3390/ijms20081944)
Supplement: Supplementary file 1 [file ijms-20-01944-s001.zip › Guenard-SuppTableS2-FC-ProbesDiffExpress.docx]

**Table S2.** Gene expression analysis for transcripts showing differential gene expression in at least one treatment.

| **Transcript** | **Gene symbol** | **Probe ID** | **BPH**  **Fold change** | **BPH**  **FDR *P* value** | **VitD_3_**  **Fold change** | **VitD_3_**  **FDR *P* value** | **VitD_3_+BPH**  **Fold change** | **VitD_3_+BPH**  **FDR *P* value** |
| --- | --- | --- | --- | --- | --- | --- | --- | --- |
| NM_001031847 | CPT1A | TC1100011395.hg.1 | 0.67 | 0.0005 | 0.67 | 2.6e-06 | 0.60 | 0.002 |
| NM_000387 | SLC25A20 | TC0300011038.hg.1 | 0.71 | 0.08 | 0.63 | 0.009 | 0.61 | 0.0002 |
| NM_001167928 | IL1RAP | TC0300009855.hg.1 | 0.81 | 0.08 | 0.83 | 0.25 | 0.78 | 0.04 |
| NM_001080392 | KIAA1147 | TC0700012836.hg.1 | 0.90 | 0.84 | 0.96 | 0.92 | 0.81 | 0.04 |
| NM_005461 | MAFB | TC2000009116.hg.1 | 1.21 | 0.35 | 1.24 | 0.25 | 1.40 | 0.04 |
| NM_020870 | SH3RF1 | TC0400012378.hg.1 | 1.33 | 0.48 | 1.62 | 0.008 | 1.47 | 0.17 |
| NM_001031700 | FAM198B | TC0400012245.hg.1 | 1.34 | 0.07 | 1.34 | 0.008 | 1.33 | 0.06 |
| NM_018404 | ADAP2 | TC1700012226.hg.1 | 1.39 | 0.08 | 1.37 | 0.008 | 1.24 | 0.21 |
| NM_000564 | IL5RA | TC0300013923.hg.1 | 0.71 | 0.07 | 0.74 | 0.008 | 0.70 | 0.06 |
| NM_001285829 | CEBPA | TC1900010386.hg.1 | 1.18 | 0.68 | 1.34 | 0.009 | 1.15 | 0.64 |
| NM_001860 | SLC31A2 | TC0900008482.hg.1 | 0.86 | 0.34 | 0.84 | 0.02 | 0.83 | 0.17 |
| NM_001271842 | SCIMP | TC1700009556.hg.1 | 1.11 | 0.62 | 1.20 | 0.02 | 1.14 | 0.51 |
| NM_016562 | TLR7 | TC0X00006625.hg.1 | 1.27 | 0.08 | 1.30 | 0.03 | 1.24 | 0.07 |
| NM_013451 | MYOF | TC1000011445.hg.1 | 1.26 | 0.42 | 1.40 | 0.03 | 1.41 | 0.07 |
| NM_004137 | KCNMB1 | TC0500012791.hg.1 | 1.21 | 0.31 | 1.24 | 0.03 | 1.18 | 0.30 |
| NM_001168357 | PLA2G7 | TC0600011953.hg.1 | 1.15 | 0.86 | 1.39 | 0.04 | 1.21 | 0.48 |
| NM_032109 | OTP | TC0500011211.hg.1 | 0.95 | 0.97 | 0.79 | 0.04 | 0.91 | 0.84 |
| NM_001122 | PLIN2 | TC0900012212.hg.1 | 0.79 | 0.07 | 0.76 | 0.04 | 0.78 | 0.08 |
| NM_000355 | TCN | TC2200009257.hg.1 | 1.21 | 0.35 | 1.30 | 0.04 | 1.15 | 0.61 |

Differential expression (Fold Changes and FDR-adjusted *P* values) for differentially expressed transcripts (time=180 vs. time=0) according to treatments. Boldface values represent statistically significant differences at FDR-adjusted *P* values ≤ 0.05. Abbreviations: FDR *P* value, False discovery rate-adjusted *P* value; BPH, Bonito fish peptide hydrolysate treatment; VitD_3_, vitamin D_3_ treatment.
