# Supplementary material for: Acute Effects of Single Doses of Bonito Fish Peptides and Vitamin D on Whole Blood Gene Expression Levels: A Randomized Controlled Trial
Source: Int J Mol Sci. 2019 Apr 20;20(8):1944. doi: 10.3390/ijms20081944 (PMC6514567; doi:10.3390/ijms20081944)
Supplement: Supplementary file 1 [file ijms-20-01944-s001.zip › Guenard-SuppTableS3-BiologicalFunctionsIPA_20190411.docx]

**Table S3.** Biological functions identified from the list of differentially expressed transcripts following VitD_3_ treatment.

| **Category** | **p-value range** | **Molecules** |
| --- | --- | --- |
| Lipid Metabolism | 1.13x10^-4^-4.95x10^-2^ | *SCIMP, CPT1A, PLA2G7, PLIN2, CEBPA* |
| Molecular Transport | 1.13x10^-4^-4.95x10^-2^ | *SCIMP, KCNMB1, SLC31A2, TLR7, CPT1A, TCN2, PLA2G7, SLC25A20, PLIN2, CEBPA* |
| Small Molecule Biochemistry | 1.13x10^-4^-4.95x10^-2^ | *SCIMP, SLC31A2, CPT1A, TCN2, PLA2G7, PLIN2, CEBPA* |
| Developmental Disorder | 3.82x10^-4^-5.3x10^-3^ | *TLR7, CPT1A, SLC25A20* |
| Hereditary Disorder | 3.82x10^-4^-1.14x10^-2^ | *CPT1A, TCN2, SLC25A20, PLA2G7, CEBPA* |
| Metabolic Disease | 3.82x10^-4^-2.8x10^-2^ | *CPT1A, TCN2, SLC25A20, PLIN2, CEBPA* |
| Organismal Injury and Abnormalities | 3.82x10^-4^-4.86x10^-2^ | *ADAP2, TLR7, MYOF, CPT1A, IL5RA, TCN2, PLA2G7, SLC25A20, PLIN2, CEBPA* |
| Cancer | 8.85x10^-4^-4.35x10^-2^ | *MYOF, TLR7, CPT1A, IL5RA, PLA2G7, CEBPA* |
| Carbohydrate Metabolism | 8.85x10^-4^-2.36x10^-2^ | *CPT1A, SLC25A20, PLA2G7, PLIN2, CEBPA* |
| Cell Cycle | 8.85x10^-4^-4.17x10^-2^ | *CEBPA* |
| Cellular Development | 8.85x10^-4^-4.93x10^-2^ | *TLR7, MYOF, OTP, IL5RA, TCN2, PLIN2, CEBPA* |
| Cellular Growth and Proliferation | 8.85x10^-4^-4.93x10^-2^ | *TLR7, MYOF, OTP, IL5RA, TCN2, PLIN2, CEBPA* |
| Connective Tissue Development and Function | 8.85x10^-4^-4.84x10^-2^ | *PLIN2, CEBPA* |
| Connective Tissue Disorders | 8.85x10^-4^-1.14x10^-2^ | *TLR7, IL5RA, PLIN2, CEBPA* |
| DNA Replication, Recombination, and Repair | 8.85x10^-4^-3.54x10^-3^ | *TLR7, CEBPA* |
| Dermatological Diseases and Conditions | 8.85x10^-4^-3.83x10^-2^ | *TLR7, IL5RA, CEBPA* |
| Digestive System Development and Function | 8.85x10^-4^-4.86x10^-2^ | *TLR7, CPT1A, PLIN2, CEBPA* |
| Embryonic Development | 8.85x10^-4^-4.93x10^-2^ | *TLR7, MYOF, OTP, IL5RA, PLA2G7, CEBPA* |
| Endocrine System Development and Function | 8.85x10^-4^-3.65x10^-2^ | *OTP, CEBPA* |
| Endocrine System Disorders | 8.85x10^-4^-6.56x10^-3^ | *CPT1A, PLIN2, CEBPA* |
| Hematological Disease | 8.85x10^-4^-4.59x10^-2^ | *MYOF, TLR7, CPT1A, IL5RA, PLA2G7, CEBPA* |
| Hematological System Development and Function | 8.85x10^-4^-4.93x10^-2^ | *TLR7, IL5RA, CEBPA* |
| Hematopoiesis | 8.85x10^-4^-4.93x10^-2^ | *TLR7, IL5RA, CEBPA* |
| Humoral Immune Response | 8.85x10^-4^-3.97x10^-2^ | *TLR7, IL5RA, CEBPA* |
| Immune Cell Trafficking | 8.85x10^-4^-3.39x10^-2^ | *TLR7, IL5RA, CEBPA* |
| Immunological Disease | 8.85x10^-4^-4.67x10^-2^ | *MYOF, TLR7, CPT1A, IL5RA, PLA2G7, CEBPA* |
| Inflammatory Disease | 8.85x10^-4^-4.86x10^-2^ | *TLR7, IL5RA, PLA2G7, CEBPA* |
| Inflammatory Response | 8.85x10^-4^-4.86x10^-2^ | *TLR7, IL5RA, CEBPA* |
| Nervous System Development and Function | 8.85x10^-4^-2.45x10^-2^ | *TLR7, OTP* |
| Nucleic Acid Metabolism | 8.85x10^-4^-5.3x10^-3^ | *CPT1A* |
| Nutritional Disease | 8.85x10^-4^-1.41x10^-2^ | *TCN2, CEBPA* |
| Organ Development | 8.85x10^-4^-4.93x10^-2^ | *MYOF, TLR7, OTP, IL5RA, CEBPA* |
| Organismal Development | 8.85x10^-4^-4.93x10^-2^ | *TLR7, MYOF, OTP, IL5RA, PLA2G7, PLIN2, CEBPA* |
| Reproductive System Disease | 8.85x10^-4^-3.65x10^-2^ | *TLR7, CPT1A, CEBPA* |
| Respiratory Disease | 8.85x10^-4^-2.88x10^-2^ | *TLR7, IL5RA, PLA2G7, CEBPA* |
| Respiratory System Development and Function | 8.85x10^-4^-2.45x10^-2^ | *TLR7, CEBPA* |
| Skeletal and Muscular Disorders | 8.85x10^-4^-1.41x10^-2^ | *TLR7, MYOF, IL5RA* |
| Tissue Development | 8.85x10^-4^-4.93x10^-2^ | *TLR7, MYOF, OTP, IL5RA, PLIN2, CEBPA* |
| Tumor Morphology | 8.85x10^-4^-3.14x10^-2^ | *TLR7, CEBPA* |
| Gastrointestinal Disease | 9.86x10^-4^-4.86x10^-2^ | *TLR7, CPT1A, IL5RA, PLA2G7, PLIN2, CEBPA* |
| Hepatic System Disease | 1.02x10^-3^-4.86x10^-2^ | *TLR7, CPT1A, PLIN2, CEBPA* |
| Lymphoid Tissue Structure and Development | 1.65x10^-3^-4.93x10^-2^ | *TLR7, IL5RA, CEBPA* |
| Cell Morphology | 1.77x10^-3^-3.48x10^-2^ | *TLR7, IL5RA, PLIN2, CEBPA* |
| Cellular Assembly and Organization | 1.77x10^-3^-4.76x10^-2^ | *MYOF, TLR7, SH3RF1, PLIN2, CEBPA* |
| Cellular Movement | 1.77x10^-3^-2.36x10^-2^ | *TLR7, IL5RA, PLA2G7, CEBPA* |
| Hepatic System Development and Function | 1.77x10^-3^-4.86x10^-2^ | *TLR7, CEBPA* |
| Hypersensitivity Response | 1.77x10^-3^-2.54x10^-2^ | *IL5RA, CEBPA* |
| Organismal Functions | 1.77x10^-3^-4.76x10^-2^ | *PLIN2, CEBPA* |
| Protein Synthesis | 1.77x10^-3^-3.87x10^-2^ | *TLR7, IL5RA, PLA2G7* |
| Skeletal and Muscular System Development and Function | 1.77x10^-3^-4.59x10^-2^ | *KCNMB1, MYOF, CEBPA* |
| Tissue Morphology | 1.77x10^-3^-4.91x10^-2^ | *MYOF, TLR7, IL5RA, PLA2G7, PLIN2, CEBPA* |
| Vitamin and Mineral Metabolism | 1.77x10^-3^-4.42x10^-2^ | *PLA2G7, PLIN2* |
| Antimicrobial Response | 2.65x10^-3^-2.65x10^-3^ | *TLR7* |
| Cell-To-Cell Signaling and Interaction | 2.65x10^-3^-4.51x10^-2^ | *KCNMB1, MYOF, TLR7, IL5RA* |
| Cardiovascular System Development and Function | 3.54x10^-3^-2.45x10^-2^ | *KCNMB1, MYOF, PLA2G7* |
| Cell Death and Survival | 3.54x10^-3^-4.73x10^-2^ | *MYOF, TLR7, IL5RA, PLA2G7, SH3RF1, CEBPA* |
| Cellular Function and Maintenance | 3.54x10^-3^-4.93x10^-2^ | *SLC31A2, MYOF, TLR7, IL5RA, CEBPA* |
| Infectious Diseases | 3.54x10^-3^-3.91x10^-2^ | *TLR7, IL5RA, PLA2G7* |
| Organ Morphology | 3.54x10^-3^-3.31x10^-2^ | *MYOF, TLR7, IL5RA, CEBPA* |
| Renal and Urological Disease | 3.54x10^-3^-1.68x10^-2^ | *TLR7, IL5RA* |
| Cardiovascular Disease | 4.42x10^-3^-3.4x10^-2^ | *KCNMB1, ADAP2, TLR7, PLA2G7, PLIN2* |
| Energy Production | 4.42x10^-3^-4.25x10^-2^ | *CPT1A* |
| Ophthalmic Disease | 4.42x10^-3^-4.42x10^-3^ | *PLA2G7* |
| Post-Translational Modification | 5.3x10^-3^-2.19x10^-2^ | *PLIN2, CEBPA* |
| Amino Acid Metabolism | 6.18x10^-3^-3.65x10^-2^ | *CEBPA* |
| Cell Signaling | 6.18x10^-3^-4.42x10^-2^ | *PLA2G7, CEBPA* |
| Drug Metabolism | 6.18x10^-3^-6.18x10^-3^ | *TCN2* |
| Reproductive System Development and Function | 6.18x10^-3^-3.05x10^-2^ | *PLA2G7, PLIN2, CEBPA* |
| Cellular Response to Therapeutics | 7.06x10^-3^-7.06x10^-3^ | *CEBPA* |
| Visual System Development and Function | 7.06x10^-3^-7.06x10^-3^ | *PLIN2* |
| Cell-mediated Immune Response | 8.66x10^-3^-4.93x10^-2^ | *TLR7, IL5RA, CEBPA* |
| Neurological Disease | 8.82x10^-3^-2.55x10^-2^ | *CPT1A, CEBPA* |
| Psychological Disorders | 8.82x10^-3^-2.55x10^-2^ | *CPT1A, CEBPA* |
| Gene Expression | 1.06x10^-2^-4.17x10^-2^ | *TLR7, CEBPA* |
| Cellular Compromise | 1.41x10^-2^-1.67x10^-2^ | *TLR7, MYOF* |
| Behavior | 2.8x10^-2^-4.35x10^-2^ | *TLR7, CPT1A, PLIN2, CEBPA* |

Ranges of *P* values and list of genes belonging to biological functions enriched among the list of differentially expressed transcripts following VitD_3_ treatment. Results were obtained using the knowledge base of the Ingenuity Pathway Analysis system®.
