# Supplementary figures and images for: Acute Effects of Single Doses of Bonito Fish Peptides and Vitamin D on Whole Blood Gene Expression Levels: A Randomized Controlled Trial
Source: Int J Mol Sci. 2019 Apr 20;20(8):1944. doi: 10.3390/ijms20081944 (PMC6514567; doi:10.3390/ijms20081944)

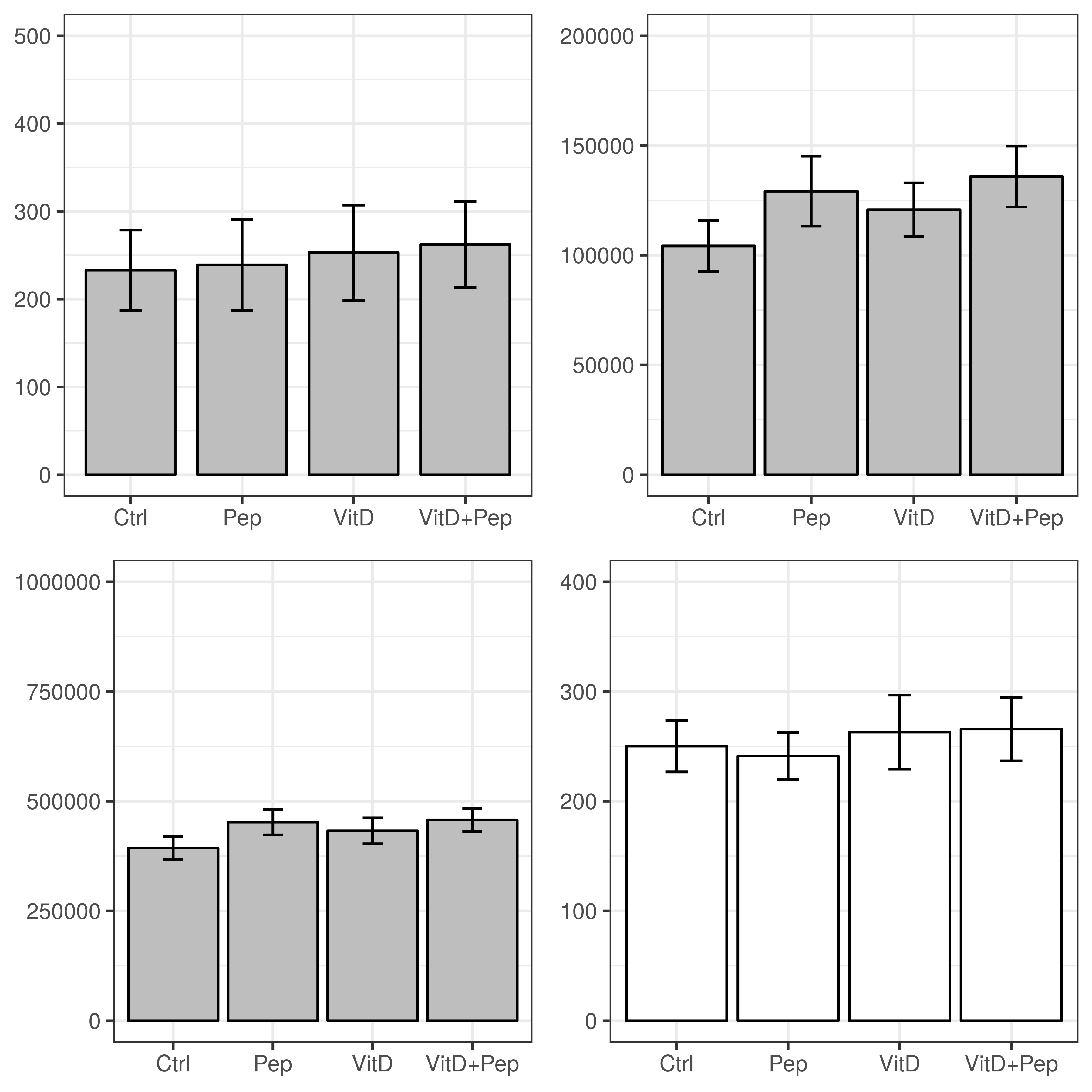

Supplement: Supplementary file 1 [file ijms-20-01944-s001.zip › Guenard-SuppFigureS1_Histo_iAUC_AUC_OGTT-montageV2.tiff]
